# Radiomics identifies distinct cortical bone texture alterations in patients with CKD using HR-pQCT

**DOI:** 10.1038/s41413-026-00515-7

**Published:** 2026-04-02

**Authors:** Youngjun Lee, Seokkyoon Hong, Miran Lee, Choongbeom Seo, Sangjun Park, Kenneth J. Lim, Sharon M. Moe, Stuart J. Warden, Rachel K. Surowiec

**Affiliations:** 1 Weldon School of Biomedical Engineering, Purdue University, West Lafayette and Indianapolis, Indiana, USA; 2 Department of Electronic Engineering, Sogang University, Seoul, South Korea; 3 Department of Total Healthcare Center, Kangbuk Samsung Hospital, Sungkyunkwan, Seoul, South Korea; 4 Department of Radiology, Seoul National University Bundang Hospital, The Seoul National University of Korea, Seoul, South Korea; 5 Department of Radiation Therapy, Samsung Medical Center, The Sungkyunkwan University of Korea, Seoul, South Korea; 6 Department of Nephrology and Hypertension, Indiana University School of Medicine, Indianapolis, Indiana, USA; 7 School of Health and Human Sciences, Indiana University Indianapolis, Indianapolis, Indiana, USA

**Keywords:** Metabolic disorders, Metabolic bone disease, Bone quality and biomechanics

## Abstract

Standard clinical imaging metrics perform poorly in predicting skeletal fragility in chronic kidney disease (CKD), particularly due to the complex and heterogeneous cortical deterioration that characterizes advanced disease. Here, this study aimed to identify radiomic features derived from high-resolution peripheral quantitative computed tomography (HR-pQCT) in tibial cortical bone that distinguish CKD-related differences and may serve as markers of subtle cortical alterations undetected by conventional imaging. HR-pQCT image stacks were obtained from 72 participants (38 non-CKD and 34 with CKD stage 5D) at 7.3% (distal) and 30% (diaphyseal) proximally from the tibial endplate, resulting in a total of 24 192 slices. In non-CKD cases, features were largely derived from first-order statistics, while complex features from higher-order statistics were more prominent in CKD cases. Although conventional HR-pQCT outcomes, such as volumetric bone mineral density, showed limited ability to differentiate CKD from non-CKD cortical bone in our population of stage 5D patients, the top features, such as Minimum and Strength, provided a significant distinction between the two groups (*P* < 0.001, Effect size *r* = from 0.813 to 0.856). Our findings demonstrate that radiomic analysis identifies cortical bone differences associated with CKD that were not distinguished by conventional HR-pQCT metrics, highlighting its potential to improve bone quality assessment in this high-risk population.

## Introduction

Individuals with chronic kidney disease (CKD) have up to 4$-$17 times higher risk of fracture, with risk increasing as kidney function declines and dialysis duration lengthens.^1–4^ Unlike other diseases that impact the skeleton, bone changes in CKD are highly heterogeneous and extend beyond osteoporosis. CKD primarily involves the cortical compartment, the dense, compact tissue forming the outer shell of long bones, where increased porosity,^5^ cortical thinning, and alterations in intracortical composition, including a reduction in bound water and changes in collagen crosslinking,^5–8^ are observed. These changes weaken the bone, making it more prone to fracture.^9^ Although trabecular bone also undergoes structural changes in CKD, such as reduced trabecular number (fewer structural elements) and increased separation, these changes contribute less to overall bone strength compared to cortical deterioration.^10,11^ While imaging outcomes such as the trabecular bone score (TBS) reflects trabecular structure and fracture risk,^12^ cortical metrics are more strongly linked to strength and fracture in CKD, and thus should be considered.^13,14^

In clinical practice, circulating biomarkers such as parathyroid hormone (PTH) and vitamin D (VitD) levels are typically evaluated first as part of the fracture risk work-up before proceeding to imaging. While dual-energy X-ray absorptiometry (DXA) is the most used imaging modality for fracture risk assessment due to its accessibility and ease, it can underestimate fracture risk in individuals with CKD.^4,15^ This is in part because DXA measures areal bone mineral density (aBMD) from two-dimensional projections and cannot assess bone quality or distinguish cortical from trabecular compartments.^6,7^ More advanced modalities such as high-resolution peripheral quantitative computed tomography (HR-pQCT), with its nominal isotropic voxel size (60-80 μm) and low effective radiation dose (3–5 μSv depending on scanner generation), enable three-dimensional evaluation of bone microarchitecture and improved sensitivity for detecting cortical deficits,^16^ even permitting estimation of bone strength through finite element analysis (FEA).^17^ HR-pQCT has therefore been used to evaluate the effects of CKD on cortical and trabecular microstructure and estimated strength,^18–20^ and more recently, time-lapse HR-pQCT has demonstrated feasibility for quantifying local bone formation and resorption in advanced CKD.^21^

Yet even with HR-pQCT, conventional cortical parameters such as cortical volumetric BMD (vBMD), cortical thickness, and porosity have shown inconsistent group differences in CKD populations.^11,19,22^ These discrepancies likely reflect the complex, heterogeneous nature of CKD-related bone disease, which involves not only microarchitectural (e.g., increased cortical porosity and trabecularization of the endosteal surface) but also matrix-level changes in mineral and collagen organization, collagen cross-linking, and bone water content, which collectively influence matrix heterogeneity and overall bone quality.^23^ Such compositional changes are not captured by standard HR-pQCT or DXA metrics, underscoring the need for complementary analytical approaches, such as radiomics, capable of quantifying cortical matrix heterogeneity from existing imaging data.

While prior studies have applied texture analysis to 2D radiographs, such images lack the resolution and depth to capture microarchitectural complexity.^24,25^ HR-pQCT offers high-resolution, 3D datasets that are well suited for radiomic extraction of cortical heterogeneity. Radiomics is a quantitative image-analysis approach that extracts a large number of first-order, texture, and higher-order statistical features from medical images to characterize tissue heterogeneity that is not apparent to visual inspection. Despite its potential to improve understanding of renal bone disease, applications of radiomics in CKD remain limited. Recent studies have explored radiomics using opportunistic clinical chest CT to evaluate bone changes in CKD, but these efforts in the vertebra have not addressed the subtle cortical microarchitectural alterations characteristic of renal disease.^26^ In our prior work, we demonstrated the feasibility of texture-based analysis, a subset of the broader radiomics framework, by extracting gray-level co-occurrence matrix (GLCM) features from HR-pQCT images of cortical bone. Using machine- and deep-learning classifiers, this pilot study achieved excellent discrimination between CKD and non-CKD groups (AUC > 0.9), providing proof of concept that texture features derived from HR-pQCT can detect CKD-related cortical alterations.^27^

Building on this foundation, the present study applies a comprehensive radiomics framework encompassing first-order, texture, and higher-order features to HR-pQCT cortical bone to identify key image-based markers that differentiate CKD from non-CKD bone. We hypothesized that radiomic analysis would reveal distinct cortical patterns reflecting microarchitectural and compositional differences beyond those captured by conventional HR-pQCT metrics. If successful, this approach, combined with machine learning, could establish non-invasive imaging biomarkers that enhance characterization of CKD-related cortical bone alterations and support future efforts toward improved fracture risk assessment.

## Results

### Participant characteristics

CKD patients were in kidney failure requiring dialysis, indicating stage 5D (on hemodialysis) disease. Patients in the dialysis group had elevated PTH levels (467.2 ± 98.9 pg/mL), while inactive vitamin D levels were within normal range (28.0 ± 2.9 ng/mL) (Table S1). Non-CKD and CKD did not differ in age, height, weight, or BMI (Table 1). Sex-stratified analyses showed that cortical area (Ct.Ar) was significantly higher in males than females in both CKD and non-CKD groups, whereas cortical vBMD and porosity did not differ by sex (Table S2). Therefore, sexes were pooled across sexes for subsequent group-level comparisons Table 1. DXA-derived aBMD and t-scores were analyzed to complement HR-pQCT findings. The mean femoral neck t-score was −0.72 ± 0.93 in non-CKD participants and −1.32 ± 1.09 in CKD participants (*P* = 0.012), confirming lower bone density in the CKD group. CKD had lower whole-body BMD z-scores than non-CKD (*P* = 0.03). Among conventional cortical outcomes measured from HR-pQCT, the only difference between groups was for cortical thickness which was 9.3% thinner in CKD (*P* = 0.02) at the tibial diaphysis but not at the distal tibial site (Table 2). Other cortical parameters including vBMD and porosity did not differ significantly between groups. When considering the addition of FEA analysis, CKD had 13.8% lower stiffness and 13.1% lower failure load compared to non-CKD (*P* = 0.016, 0.019) at the distal site only. Shapiro-Wilks results indicated that data from all groups exhibited a normal distribution (*P* > 0.05).

**Table 1:** Participant characteristics

Characteristics	Mean (standard deviation), *n* (%)		*P*-value
	Non-CKD (*n* = 38)	CKD (*n* = 34)	
Age/year	54 ± 14.70	52.34 ± 13.35	0.58
Sex (Female)	18 (47%)	14 (41%)	0.79
Height/cm	171.22 ± 10.18	169 ± 11.00	0.61
Weight/kg	79.06 ± 14.70	84.06 ± 17.55	0.20
BMI /(kg/m^2^)	26.84 ± 3.76	29.26 ± 6.58	0.07
Whole body aBMD	0.980 ± 0.128	0.976 ± 0.129	0.197
Whole body z-score	0.39 ± 1.14	−0.30 ± 1.17	0.009
Whole body t-score	0.03 ± 1.24	0.74 ± 1.48	0.021
Femoral neck aBMD	0.834 ± 0.124	0.793 ± 0.153	0.211
Femoral neck z-score	0.19 ± 0.85	−0.49 ± 0.97	0.002
Femoral neck t-score	−0.72 ± 0.93	−1.32 ± 1.09	0.012

The *P*-values between non-chronic kidney disease (non-CKD) and CKD patients were determined utilizing *t*-tests or chi-squared tests. Values are shown as means ± standard deviation or as counts (percentages). *BMI* body mass index, *BMD* bone mineral density

**Table 2:** Conventional HR-pQCT cortical outcomes

Bone	Location	Cortical Outcome	Mean ± Standard Deviation		Value	
			Non-CKD	CKD	*P*-value	Cohen’s *d*
Tibia	Distal	Ct.vBMD/(mgHA/cm^3^)	892.34 ± 73.65	862.24 ± 94.18	0.163	0.360
		Ct.Ar/mm	145.51 ± 35.36	132.36 ± 30.36	0.126	0.396
		Ct.Th/mm	1.59 ± 0.35	1.47 ± 0.31	0.158	0.365
		Ct.Pm/mm	108.91 ± 12.48	108.85 ± 11.58	0.985	0.005
		Ct.Po/%	3.09 ± 1.60	3.09 ± 1.55	0.997	0.001
		Ct.PoDm/mm	0.24 ± 0.03	0.22 ± 0.03	0.064	0.481
		Ct. Stiff. Dist/(kN/mm)	95.96 ± 28.34	94.36 ± 26.49	0.807	0.058
		Ct. Failure Load. Dist/kN	8.24 ± 2.39	8.17 ± 2.27	0.906	0.028
		Ct. Stiff. Prox/(kN/mm)	148.97 ± 37.48	128.37 ± 31.93	0.016	0.588
		Ct. Failure Load. Prox/kN	12.78 ± 3.11	11.11 ± 2.71	0.019	0.571
	Diaphyseal	Ct.vBMD/(mgHA/cm^3^)	1 027.43 ± 23.80	1 011.86 ± 45.75	0.095	0.440
		Ct.Ar/mm	295.02 ± 50.06	283.06 ± 89.49	0.408	0.217
		Ct.Th/mm	6.00 ± 0.81	5.44 ± 1.03	0.023	0.608
		Ct.Pm/mm	80.68 ± 7.21	82.28 ± 8.46	0.435	0.204
		Ct.Po/%	1.02 ± 0.52	1.23 ± 0.98	0.298	0.273
		Ct.PoDm/mm	0.22 ± 0.03	0.21 ± 0.04	0.089	0.449
		Ct. Stiff. Dist/(kN/mm)	301.45 ± 53.98	288.37 ± 64.62	0.325	0.256
		Ct. Failure Load. Dist/kN	26.10 ± 4.68	24.85 ± 5.72	0.351	0.242
		Ct. Stiff. Prox/(kN/mm)	300.95 ± 53.70	287.10 ± 64.81	0.365	0.235
		Ct. Failure Load. Prox/kN	26.06 ± 4.66	24.91 ± 5.74	0.393	0.222

Comparison of the conventional cortical HR-pQCT measures between the non-CKD and CKD groups’ results. ± < 0.05 means statistical significance. µFE outcomes of stiffness and failure load considered both trabecular and cortical bone compartments. *Ct.vBMD* cortical volumetric bone mineral density, *Ct.Ar* cortical area, *Ct.Th* cortical thickness, *Ct.Pm* cortical perimeter, *Ct.Po* cortical porosity, *Ct.PoDm* cortical pore diameter, *Ct.Stiff.dist* cortical distal total stiffness, *Ct.Fload.dist* cortical distal total failure load, *Ct.Stiff.prox (kN)* cortical proximal total stiffness, *Ct.Fload.prox (kN)* cortical proximal total failure load

### Radiomic feature selection and top-ranked features

**Fig. 1:**
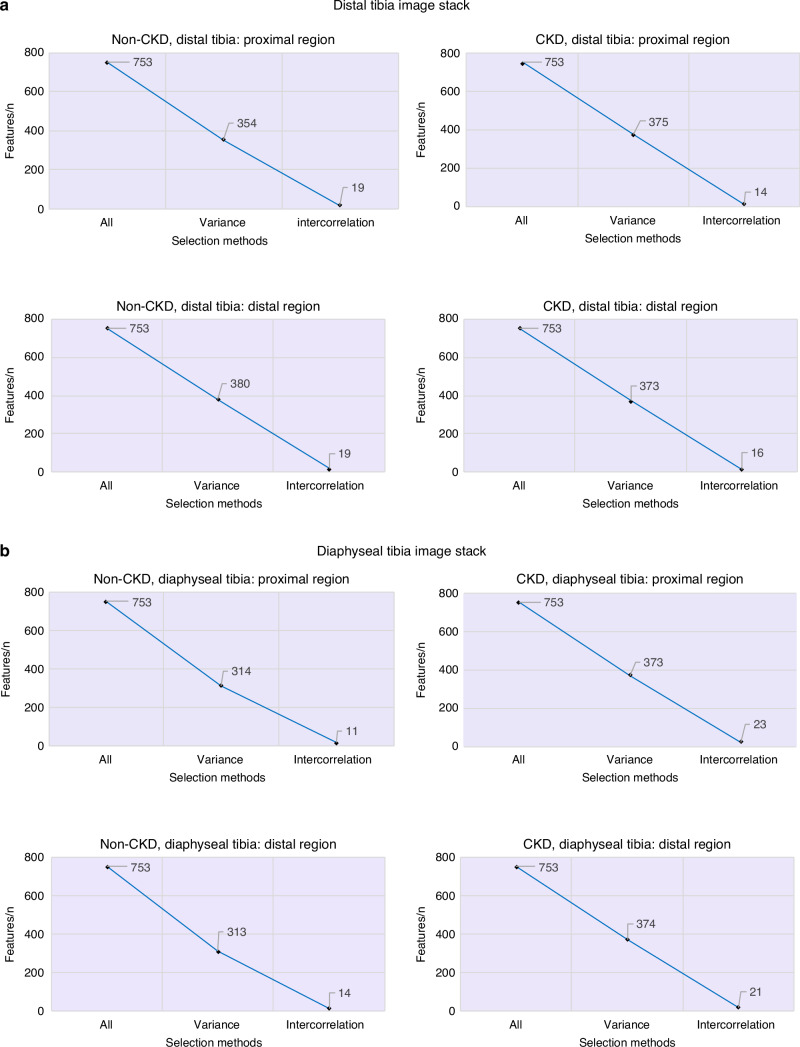
Feature selection using variance and intercorrelation analysis was performed at the distal (**a**) and diaphyseal (**b**) sites at the proximal and distal subregions (depicted in Fig. 6b) of the tibia. After removing all features with low variance (≤0.20) and high inter-correlation (≥0.80), the final selected features were those with sufficient variance and low intercorrelation. The number of features meeting the selection process is depicted in the bottom right-hand corner of each plot

**Fig. 2:**
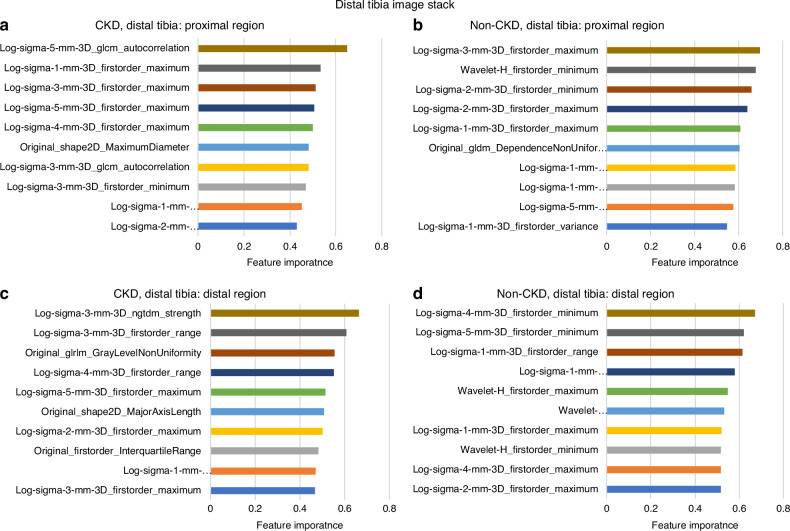
Top 10 ranked features from distal tibia image stack. After excluding low variance and highly inter-correlated features, the remaining features were normalized and ranked by importance, with values closer to 1.0 indicating higher importance and values closer to 0 indicating lower importance. (**a**): CKD, proximal subregion, (**b**): Non-CKD, proximal subregion, (**c**): CKD, distal subregion, (**d**): Non-CKD, distal subregion

**Fig. 3:**
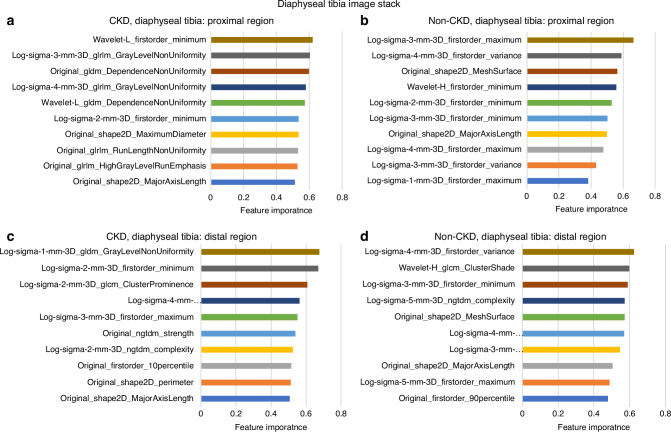
Top 10 ranked features from diaphyseal tibia image stack. After excluding low variance and highly inter-correlated features, the remaining features were normalized and ranked by importance, with values closer to 1.0 indicating higher importance and values closer to 0 indicating lower importance. (**a**): CKD, proximal subregion, (**b**): Non-CKD, proximal subregion, (**c**): CKD, distal subregion, (**d**): Non-CKD, distal subregion

A bin width of 0.2 yielded the best feature extraction results. From both the distal and diaphyseal tibia in CKD and non-CKD image stacks, 753 features were extracted across the proximal and distal subregions. After filtering using optimized thresholds for low variance (below 0.2) and highly inter-correlated features (above 0.8), 11 to 23 key features were selected per subregion per tibial site (Table S3). Figure 1a, b show the remaining features after this process. A full list of selected features, normalized and ranked by average, is provided in Figs. 2 and 3. The top five features with the highest feature importance per subregion for CKD and non-CKD are presented below. Radiomic feature importance rankings were determined separately for CKD and non-CKD groups within each subregion (proximal and distal) of the distal and diaphyseal tibia. The top features for each group are described below and illustrated in Figs. 2, 3. For each feature, the parenthetical terms denote the feature class (e.g., first-order, GLCM), the filter type (e.g., Laplacian of Gaussian or wavelet), and, where applicable, the filter parameter (e.g., σ = 1–5), which specifies the level of image smoothing applied prior to feature extraction.

#### Distal Tibia

##### Proximal Subregion

For CKD (Fig. 2a), the top five ranked features were Autocorrelation (GLCM, sigma = 5; a measure of how voxel intensities are correlated with their neighbors, reflecting texture regularity) and Maximum (first-order; the highest voxel intensity, reflecting regions of greater mineralization) derived from Log-filtered images (sigma = 1, 3, 4, and 5). For non-CKD (Fig. 2b), top five features included: Maximum (First order) from Log-filtered images (sigma = 3); Minimum (first-order; lowest voxel intensity, corresponding to less mineralized or low-density regions) from wavelet-transformed images (high-pass filter); Minimum (first-order) derived from Log-filtered images (sigma = 2); and Maximum (first-order) derived from Log-filtered images (sigma = 1 and 2).

##### Distal Subregion

For CKD (Fig. 2c), the top five ranked features were Strength (Neighboring Gray Tone Difference Matrix, NGTDM; quantifies the overall intensity strength of neighboring voxels, with higher values reflecting greater texture heterogeneity) and Range (first-order; the difference between maximum and minimum voxel intensities, reflecting intensity spread) both derived from Log-filtered images (sigma = 3). Additional top features included Gray Level Non-Uniformity (GLRLM; a measure of irregularity in gray-level distribution, with higher values indicating less uniform density) from the original images, as well as Range (First order, sigma=1) and Maximum (First order, sigma = 5) from Log-filtered images. For non-CKD (Fig. 2d), the top five features included Minimum (First order) from Log-filtered images sigma = 4 and sigma = 5; Range (First order) and Interquartile Range (First order; the difference between the 75th and 25th percentile voxel intensities, reflecting mid-range density variation) from Log-filtered images (sigma = 1); and Maximum (First order) derived from wavelet-transformed images (high-pass filter).

#### Diaphyseal Tibia

##### Proximal Subregion

For CKD (Fig. 3a), the top five ranked features included Minimum (first-order) from wavelet-transformed images (low-pass filter); Gray Level Non-Uniformity (GLRLM) from Log-filtered images (sigma = 3, 4); and Dependence Non-Uniformity (GLDM; quantifies non-uniformity in voxel dependence, reflecting heterogeneity) from both original and wavelet-transformed images (low-pass filter). For non-CKD (Fig. 3b), the top five features were Maximum (first-order) from Log-filtered images (sigma = 3); Variance (first-order; quantifies the spread of voxel intensities) from Log-filtered images (sigma = 4); Mesh Surface (2D shape-based; quantifies the surface area of the cortical mask) from the original images; and Minimum (first-order) from both wavelet-transformed images (high-pass filter) and Log-filtered images (sigma = 2).

##### Distal Subregion

The top five ranked features for CKD (Fig. 3c) were all from log filtered images and included Gray Level Non-Uniformity (GLDM, sigma = 1), Minimum (first-order, sigma = 2), Cluster Prominence (GLCM, sigma = 2; measures the peakedness of gray-level clusters), Long Run High Gray Level Emphasis (GLRLM, sigma = 4; captures long consecutive runs of high-intensity voxels, reflecting denser regions), and Maximum (first-order, sigma = 3). For non-CKD (Fig. 3d), the five top features were Variance (first-order) from Log-filtered images (sigma = 4); Cluster Shade (GLCM) from wavelet-transformed images (high-pass filter); Minimum (first-order) from Log-filtered images (sigma = 3); Complexity (NGTDM; measures the complexity of texture patterns) from Log-filtered images (sigma = 5); and Mesh Surface (2D shape-based) from the original images.

In summary, when comparing across anatomical sites, CKD bone at the distal tibia was predominantly characterized by Autocorrelation and Strength, whereas diaphyseal CKD bone was defined by Minimum and Gray Level Non-Uniformity, indicating site-specific textural heterogeneity. In contrast, non-CKD bone showed greater consistency across sites, with Maximum, Minimum, and Variance features repeatedly emerging as top-ranked descriptors.

##### Validation of Feature Independence and Correlation Analysis

Heatmaps of all the top-ranked features showed minimal inter-correlation, confirming feature independence and robustness (Figs. S1–4). The correlation coefficient distribution was centered near zero, with most values ranging between −0.75 and 0.75, further supporting low redundancy among extracted features (Figs. S5 and S6).

To examine the relationship between radiomic texture and estimated mechanical properties from HR-pQCT, we correlated the two most discriminative features in CKD, Strength (NGTDM) and Minimum (first-order) with µFE outcomes. No significant correlations were observed between either feature and µFE stiffness or failure load (Tables S4, S5). Correlation coefficients were low in magnitude ( | r | < 0.30), with weak negative trends for Strength and weak positive trends for Minimum, consistent with the possibility that greater cortical textural heterogeneity is associated with reduced mechanical competence.

##### Discriminating CKD vs. Non-CKD Using Top Radiomic Features

For the proximal region of the distal tibia, where both CKD ($P$ = 0.600) and non-CKD ($P$ = 0.176) groups for the Strength feature met the assumption of normality, a t-test was applied and showed no significant difference between groups ($P$ < 0.434, Cohen’s d = 0.251 [95% CI (−0.373, 0.872)]. For all other subregions that did not satisfy normality ($P$-values ranging from 0.002 to 0.045), the Mann-Whitney U test was used, revealing statistically and practically significant differences between CKD and non-CKD groups for both the Strength and Minimum features ($P$ < 0.001, Effect size $r$ = from 0.813 to 0.856).

**Fig. 4:**
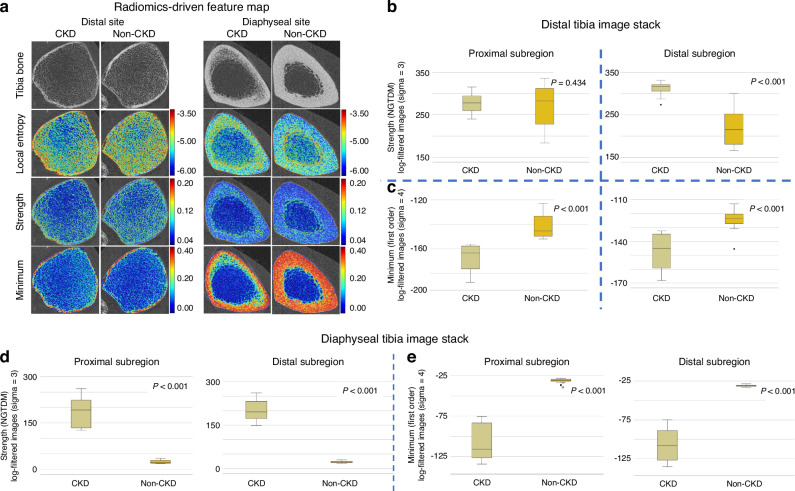
Radiomics-based qualitative and quantitative assessment of cortical bone heterogeneity in CKD. **a** Radiomic feature maps show differences in heterogeneity of bone texture between CKD and non-CKD tibial HR-pQCT images. The local entropy feature highlights areas with varying degrees of texture complexity, with higher values indicating regions of more complex structure. In CKD, one of the top-ranked features is NGTDM-Strength, which indicates the degree of heterogeneity. In non-CKD, one of the most important features is First Order-Minimum, which focuses on regions with low signal intensities. Values, closer to red, indicate areas with more intense signals based on the feature being overlayed. Box plots demonstrating the average NGTDM-Strength (**b**) and First Order-Minimum (**c**) in the distal tibial image stack in the proximal (left) and distal (right) subregions, respectively. In general, CKD bone showed higher NGTDM-Strength and lower First Order-Minimum compared to non-CKD cortical bone. Similar results were seen at the diaphyseal tibia stack for NGTDM-Strength (**d**) and First Order-Minimum (**e**) but with less variability than the distal image stack. *P*-values from *t*-tests or Mann Whitney-U tests (when distributions were non-normal) are shown on the figures

As shown in Fig. 4a–e, the features chosen for evaluation were selected based on their ability to capture key aspects of bone texture in CKD and non-CKD cortical bone. Radiomic maps in Fig. 4a illustrate that Entropy, Strength, and Minimum highlight different aspects of cortical heterogeneity. Entropy, which quantifies textural complexity, showed higher values (red/yellow) in CKD bone, consistent with increased cortical disorganization. The NGTDM-Strength feature (Fig. 4a), the top-ranked feature for CKD cortical bone at the distal tibia (Fig. 2c), measures the intensity of local texture strength, particularly in irregular and complex regions. This feature was significantly higher in CKD compared to non-CKD at the distal subregion ($P$ < 0.001) but not the proximal subregion (normal distribution, *P* = 0.434, Cohen’s d = 0.251 [95% CI (−0.373, 0.872)] (Fig. 4b). For the diaphyseal stack, NGTDM-Strength was significantly higher in CKD vs. non-CKD in both subregions (both $P$ < 0.001).

The Minimum feature (Fig. 4a, c, e), a top radiomics feature in non-CKD cortical bone (Fig. 2b, d), reflects the ability to capture areas of low intensity associated with more homogeneous bone tissue. Non-CKD cases exhibited higher values for this feature compared to CKD in the distal stack (Fig. 4c, proximal and distal subregions both *P* < 0.001) and diaphyseal stack (Fig. 4e, proximal and distal subregions both *P* < 0.001). All subregions aside from the proximal subregion of the distal image stack did not satisfy normal distribution thus the Mann-Whitney U-test was used and effect sizes ranged from 0.813 to 0.856).

**Fig. 5:**
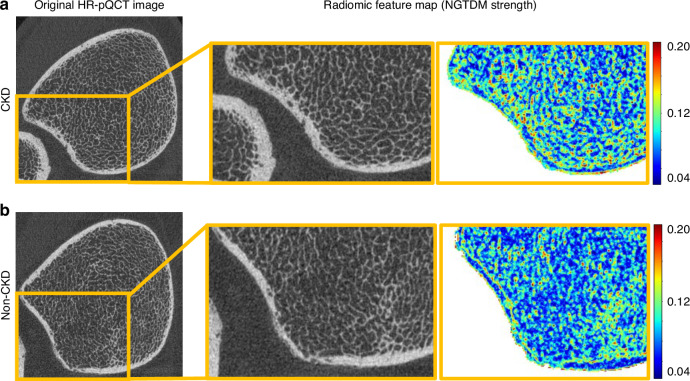
Visualization of the radiomic Strength feature in CKD and non-CKD cortical bone. Radiomics maps based on the Strength feature demonstrate differences in cortical texture between CKD and non-CKD. **a**, **b** Representative distal tibia HR-pQCT images were selected based on overlapping conventional cortical parameters illustrating cases in which standard measures alone do not clearly differentiate CKD from non-CKD bone: (CKD stage 5D: Ct.Ar = 127.5 mm², Ct.vBMD = 884.4 mgHA/cm³, Ct.Po = 2.6%; non-CKD: Ct.Ar = 138.5 mm², Ct.vBMD = 906.4 mgHA/cm³, Ct.Po = 2.4%). Corresponding radiomic Strength maps highlight spatial texture differences indicative of increased cortical heterogeneity in CKD bone compared with non-CKD

Based on the matched conventional HR-pQCT parameters at the distal tibia (CKD stage 5D: Ct.Ar = 127.5 mm², Ct.vBMD = 884.4 mgHA/cm³, Ct.Po = 2.6%; non-CKD: Ct.Ar = 138.5 mm², Ct.vBMD = 906.4 mgHA/cm³, Ct.Po = 2.4%), representative cases were selected to illustrate that conventional cortical measures can appear comparable between CKD and non-CKD bone (Fig. 5a, b). Despite similar conventional measures, these radiomic differences capture textural alterations beyond what is reflected by cortical porosity and are not qualitatively apparent in the original HR-pQCT images.

## Discussion

Our findings highlight distinct radiomic features of tibial cortical bone in individuals with CKD stage 5D, which were notably heterogeneous across subregions. Conventional HR-pQCT outcomes, such as vBMD, cortical thickness, and porosity, were limited in distinguishing between CKD and non-CKD, with only modest differences in stiffness and failure load from µFE. In contrast, top radiomic features demonstrated stronger discrimination with larger effect sizes, supporting future machine learning model development defining the radiomic bone phenotype in CKD.

Radiomics revealed heterogeneous texture alterations in CKD bone. For example, in non-CKD bones, top features were primarily derived from first-order statistics, reflecting basic intensity distribution, while CKD features were derived from higher-order statistics, consistent with alterations in microarchitecture and organization. Specifically, the “first-order minimum” intensity, representing the lowest voxel intensity in a given region, was lower in CKD bone across all tibial subregions, suggesting less dense tissue, potentially due to porous or degraded bone. These findings align with Wang et al.^28^ who demonstrated lower maximum intensity in osteoporotic bones on dual-energy CT, linking this to increased resorption and reduced mineral content. Although the original HR-pQCT images showed no obvious visual differences between CKD and non-CKD samples, the radiomic map revealed higher cortical heterogeneity in CKD stage 5D. This aligns with expected pathophysiological changes such as increased porosity and disrupted intracortical organization. Such findings support the biological relevance of radiomic features in detecting early cortical deterioration that may be overlooked by conventional assessments. Notably, Ct.Po derived from conventional HR-pQCT analysis did not differ significantly between groups (Table 2). However, the radiomics approach captured broader textural heterogeneity within the cortical compartment. This may reflect the combined influence of intracortical remodeling, subtle trabecularization at the endosteal surface, and spatial variations in mineral or matrix density that are not resolved by threshold-based porosity metrics. Because the cortical segmentation included small intracortical pores and transitional regions, the extracted features likely encode both porosity-related changes and other microarchitectural variations, which could explain why radiomics distinguished CKD from non-CKD bone even when Ct.Po did not.

In CKD, top radiomics features, such as NGTDM strength, were derived from advanced statistical methods (GLCM, GLRLM, GLDM, NGTDM), capturing subtle textural variations. NGTDM strength, which measures contrast in relation to neighboring voxels, was higher in the distal subregion of the distal tibia and all diaphyseal subregions, suggesting more heterogeneous tissue in CKD bone, potentially linked to compromised bone quality. These findings expand upon earlier studies, such as those associating GLCM-based features with osteoporotic changes, further supporting the clinical relevance of subregional texture analysis in CKD bone.^29,30^ Importantly, our feature selection focused on robust metrics with low variation, utilizing an automated deep-learning pipeline for cortex segmentation, enhancing reproducibility, and reducing manual segmentation error or need for manual correction.^27^ This is crucial for clinical and research applications, as previous studies have shown that radiomic features can vary based on imaging parameters, including exposure settings (kV, mAs), filter type, and imaging distance, underscoring the importance of robust feature selection in radiomics.^31^

Subregional analysis of tibial stacks further illustrates that key radiomic features varied within the same image stack. For example, Autocorrelation (GLCM) and Strength (NGTDM) characterized CKD bone in the distal tibia, while Maximum and Minimum (first-order) features were more prominent in non-CKD bone. At the diaphyseal tibia, CKD features included Minimum (first-order) and Gray Level Non-Uniformity (GLDM), whereas non-CKD bone was characterized by Maximum and Variance (first-order). Such site-specific patterns may reflect differences in cortical organization and loading environment between distal and diaphyseal regions, factors that could contribute to their differing fracture susceptibility. Radiomic signatures appeared more consistent at the diaphyseal site, which may relate to its thicker, more uniform cortex and reduced partial-volume effects relative to the distal tibia with its thinner cortices. This observation suggests that the diaphysis could provide a more stable region for radiomic evaluation, although validation in larger, multi-site studies is needed to confirm reproducibility.

Correlation analysis showed that Strength and Minimum, while top radiomic features able to significantly distinguish CKD vs. non-CKD cortical bone, were not significantly correlated with µFE-derived stiffness or failure load. This may suggest that these radiomic features may be sensitive to compositional or matrix-level alterations that are not reflected in bulk mechanical estimates. This interpretation aligns with prior work showing that tissue compositional changes, such as altered mineralization, collagen cross-linking, or bound water loss, can degrade bone quality independently of apparent stiffness.^32–34^ Importantly, stiffness and failure load were the only HR-pQCT outcomes that differed significantly between CKD and non-CKD at the distal tibia (Table 2). However, these µFE-based estimates reflect only the apparent, pre-yield mechanical behavior and do not account for post-yield or failure characteristics such as toughness or energy dissipation. Future studies should therefore test whether top radiomic features correlate with post-yield properties, alongside compositional measures of collagen, mineral, and water content obtained from spectroscopy or magnetic resonance imaging to better define their biological basis. Such multimodal validation would further clarify whether radiomics can detect composition-related cortical deficits that are not captured by density- or stiffness-based measures in CKD.

Our findings align with previous findings on altered bone in CKD,^4,8^ but extend these insights by characterizing texture changes not evident through conventional analysis. This approach is consistent with broader radiomics applications, where it has successfully captured subtle tissue changes in tumors,^35^ lungs,^36^ brain tissue,^37^ and bone.^38^ For example, a recent study achieved 94% accuracy in classifying osteoporosis using radiomics from dual-energy CT spine images.^39^ To our knowledge, this study is the first to apply radiomics to HR-pQCT imaging for cortical bone analysis. Previous research has applied textural analysis to trabecular bone, originating from the trabecular bone score (TBS) in DXA, now an additional osteoporosis management tool.^40^ Similarly, the distinct radiomic features identified in CKD bone may serve as non-invasive biomarkers, addressing limitations of DXA in assessing CKD bone quality.^41–43^

Limitations of our study include the single-institution sample design, which may introduce bias. Future studies should validate these findings in larger, multi-center, prospective cohorts across a broader range of CKD stages. Model development deliberately began with advanced disease stages to first confirm that radiomic features can reliably distinguish clearly established cortical abnormalities before extending application to earlier and more subtle disease stages. This stepwise approach was informed by our previous pilot study, in which advanced CKD at the tibial sites, particularly the diaphyseal region, demonstrated excellent classification performance (AUC = 0.99, F1 = 0.99 in test data; AUC = 0.94, F1 = 0.96 in independent validation.^27^ Therefore, future studies should include earlier CKD stages to validate the robustness of radiomic features and evaluate their potential utility in guiding preventive treatment strategies. HR-pQCT is currently limited to certain research centers; however, the radiomics pipeline developed in this study is modality-agnostic and could be adapted to emerging imaging platforms. In particular, photon-counting CT (PCCT), which is becoming increasingly available and offers spatial resolution approaching that of HR-pQCT, may provide a clinically scalable alternative for radiomics-based cortical bone assessment. Further exploration of the biological basis of these radiomic features, including their associations with mineral, collagen, and water content, could provide new insights into CKD-related bone pathology.

Although prior studies have frequently reported higher cortical porosity in CKD compared with controls, our cohort did not demonstrate statistically significant group differences. This may reflect the limited sample size, the heterogeneity of CKD-related bone disease, and the relatively higher BMI in the CKD group, which could confer partial protective mechanical loading and attenuate porosity differences. Evidence from a recent twin study by Nissen et al. supports this interpretation, showing that individuals with higher body weight exhibit lower cortical porosity and greater cortical cross-sectional area due to increased mechanical loading.^44^ Age-related effects may have also contributed; reference data from Warden et al. indicate that tibial cortical porosity remains low before age 50 and increases thereafter, with typical adult values between 2% and 5%.^45^ Given the mean participant age ( ~ 55 years), both groups likely fell near the upper range of normative expectations.

The absence of uniformly collected circulating PTH and vitamin D data limited our ability to assess direct associations with HR-pQCT outcomes (including Ct.Po) or radiomic features. PTH and 25(OH)D levels were obtained retrospectively via chart review, with timing relative to HR-pQCT imaging ranging from a few days to nearly one year, which restricts interpretability. Among dialysis patients, the average PTH level was 467.2 ± 98.9 pg/mL. This falls within the broader 2017 KDIGO recommended range of approximately 2–9 times the assay-specific upper normal limit (typically ∼130–600 pg/mL),^46–48^ yet exceeds the more conservative K/DOQI 2003 target range of 150–300 pg/mL^49^ or CKD stage 5D. Also, laboratory assessments were not temporally aligned with HR-pQCT imaging; correlation analyses between biochemical markers and imaging-derived features were not performed. Future studies incorporating prospective, same-day serum collection at the time of imaging will enable direct, temporally aligned assessment of biochemical and imaging markers of bone quality.

To reduce computational burden and capture subregional variation, radiomic features were extracted from proximal and distal 10-slice subregions rather than full 168-slice stacks. While this approach enabled targeted evaluation of cortical heterogeneity, it may not reflect all within-stack variation; future studies should examine whole-stack or slice-wise analyses. Additionally, this study focused on the tibia, a clinically relevant weight-bearing site where fractures are more commonly observed in advanced CKD patients.^50^ The radius, as a non–weight-bearing site, may exhibit different patterns of cortical changes, and future studies should include both sites to delineate skeletal site–specific effects. Moreover, while raw values were reported in the present study given the age- and sex-matched design, future work should incorporate Z-score–based approaches to provide additional normalization and further validate conventional outcomes.

In summary, our study demonstrates the potential of radiomics to provide a detailed assessment of CKD-related bone changes. By identifying distinct texture features between the proximal and distal subregions within the same tibial stack, the analysis distinguished CKD stage 5D from non-CKD cortical bone even when conventional HR-pQCT outcomes showed limited group differences. These findings suggest that radiomic analysis can capture subtle spatial heterogeneity not reflected by density-based measures. With further validation, this approach could complement existing imaging methods and support the development of machine learning models to improve fracture risk assessment and management in CKD.

## Methods

### Study design

**Fig. 6:**
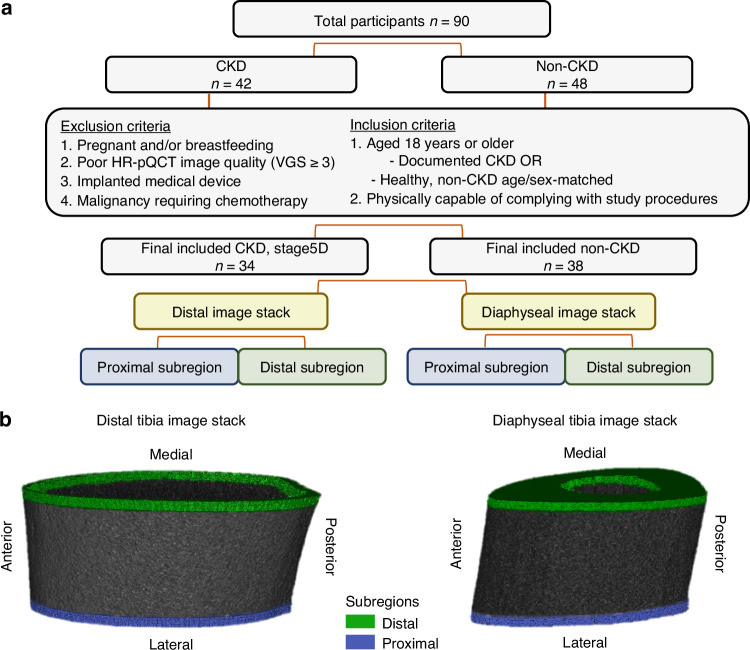
Study workflow and tibial subregion definition for radiomics analysis. **a** Flow diagram for study inclusion and (**b**) 3D cross-sectional rendering of the distal and diaphyseal tibial image stack with the analysis subregions comprised of 10 consecutive slices depicted in green and blue for the distal and proximal subregions, respectively

We retrospectively identified 72 adults (34 CKD stage 5D and 38 age- and sex-matched non-CKD) who had undergone HR-pQCT imaging within the Function, Imaging, and Testing Resource Core of the Indiana Center for Musculoskeletal Health between 2018 and 2024 (Fig. 6a). Institutional Review Board approval (Study #170755085) was obtained, and all participants provided written informed consent for their images and data to be utilized. To be included in the current analyses, participants needed to have documented CKD (CKD cohort) or no known conditions affecting musculoskeletal health (non-CKD cohort) and HR-pQCT images with a motion artifact of ≤ 3.^51^ Circulating PTH and vitamin D values were obtained through retrospective chart review of clinical laboratory data within each participant’s electronic medical record. This data was not available for the age- and sex-matched non-CKD controls and thus only PTH and VitD for the CKD patients were reported.

#### High-resolution Peripheral Quantitative Computed Tomography (HR-pQCT)

HR-pQCT images of the participant’s leg on the same side as their dominant arm were acquired using an XtremeCT II (Scanco Medical, Bruttisellen, Switzerland) at 7.3% (distal) and 30% (diaphyseal) of tibial length proximal from the distal tibia joint surface, as we have previously described.^52^ The scanner operated at 68 kVp and 1.47 mA, producing 168 slices at a 60.7 μm voxel size. A manufacturer-supplied phantom was used to monitor scanner performance throughout the study. Cortical bone volumetric bone mineral density, area, thickness, and percent porosity were calculated using manufacturer algorithms. Bone stiffness and failure load for the cortical region was estimated through micro-finite element (µFE) analysis (Scanco Medical FE software v1.13), simulating axial compression (See Supplemental 1.1). These simulations assume linear elastic, isotropic material behavior and small deformation, thus representing the apparent pre-yield mechanical response rather than post-yield or failure properties.

### Source model-based acceleration for segmentation

We utilized a pre-trained segmentation model based on U-net architecture, as detailed by our prior study, which was initially trained on distal and diaphyseal tibial images (source model).^27^ Additional training with distal and diaphyseal tibia images further accelerated the pre-trained network (transfer learning), following gray-level normalization and discretization (detailed below). This process enabled analysis of the gray-level range and isolation of cortical bone from soft tissue. After transfer learning, each image stack was divided into two equal subregions of ten consecutive slices for proximal and distal analysis at each site (diaphyseal, distal) (Fig. 6b). This subregion analysis was done to evaluate the presence of heterogeneity across the bone within a typical HR-pQCT imaging stack.

### Feature extraction

Image preprocessing and feature extraction were performed using PyRadiomics (v.3.1.0).^53^ Regarding preprocessing, image resampling (in-plane resolution 0.06 × 0.06 mm) was performed according to current guidelines.^54^ Grey-level normalization and discretization were done as follows: first, the grey-level pixel intensities were standardized using z-score normalization. Next, the standardized values were scaled to the range 0-1 using min-max normalization. After this process, exploratory extraction of the primary parameters (i.e., gray-level ranges) was performed only on the training set to avoid information leakage from the test set to determine the appropriate bin width for discretization. In this step, we analyzed the gray-level range of the normalized image using candidate bins of 0.2, 0.3, 0.4, and 0.5. Based on this evaluation, a fixed bin width of 0.2 was selected, consistent with the Image Biomarker Standardization Initiative (IBSI),^54^ which recommends a bin width in the range of 0.1–0.5 to balance noise suppression and preservation of heterogeneity.^53,55^ In addition to the original images, features were also extracted from the filtered image processed using Laplacian of Gaussian filters (sigma = 1, 2, 3, 4, 5) and wavelet decomposition. All available histogram-based first-, second- (texture), and higher-order features were extracted, and are described in detail in the official PyRadiomics documentation^53^

### Feature Selection

**Fig. 7:**
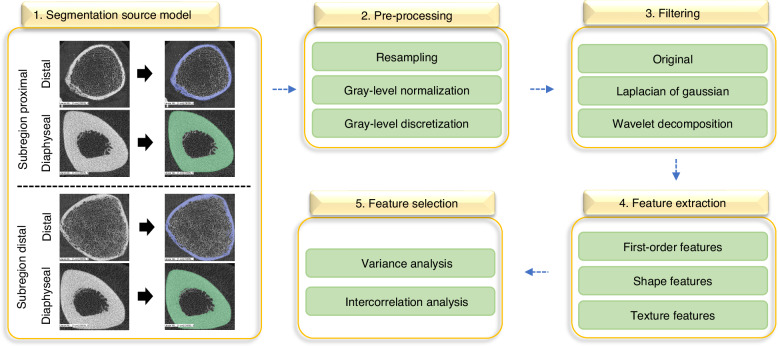
Radiomics-driven HR-pQCT texture analysis workflow

The Python *scikit-learn* package was used for feature selection.^56^ Feature selection was conducted independently for each region through a multi-step process to eliminate redundant or irrelevant features. Feature filtering was systematically evaluated across different thresholds (e.g., variance < 0.1–0.5; Pearson correlation coefficient > 0.5–0.9) across repeated iterations to identify stable, non-redundant features. The complete analysis pipeline is illustrated in Fig. 7 and summarized in Table S3.

### Statistical Analysis

Continuous data are presented as median and interquartile (1^st^–3^rd^) range and categorical data as counts and proportions of values. Data preparation was carried out utilizing the *pandas* Python library.^57^ Heatmaps and histograms were used to validate the feature correlations, visualize feature distributions, and confirm that most features exhibited low inter-correlation. Correlation analysis between radiomic features and µFE outcomes were performed across all participants, regardless of CKD status, to explore potential relationships. Stiffness and failure load were selected as comparators, as they are validated surrogates of bone strength and standard HR-pQCT outputs from SCANCO.^58,59^ For visualization, radiomics-driven, color-coded feature maps were overlaid onto the original HR-pQCT image stack. Box plots of several key selected features were generated to assess their ability to differentiate between CKD and non-CKD cortical bone. The Shapiro-Wilk test was used to assess data normality (*P* > 0.05 indicating normality). Group differences were tested using two sample *t*-tests (with Cohen’s *d* for effect size). For non-normal data, Mann-Whitney *U* test (with *r* for effect size) was used.^60^ Participant characteristics and conventional cortical HR-pQCT outcomes are presented as mean ± standard deviation and compared using the same procedures. Significance was set at *P* ≤ 0.05 using SPSS v28 (IBM Corp., Armonk, NY).

## Supplementary information

Clean version-Supplemental files

## Data Availability

The data that support the findings of this study are available from the corresponding author upon reasonable request.
