# Supplementary material for: Radiomics identifies distinct cortical bone texture alterations in patients with CKD using HR-pQCT
Source: Bone Res. 2026 Apr 2;14:36. doi: 10.1038/s41413-026-00515-7 (PMC13046811; doi:10.1038/s41413-026-00515-7)
Supplement: Supplementary file 1 — Clean version-Supplemental files [file 41413_2026_515_MOESM1_ESM.docx]

**Radiomics Identifies Distinct Cortical Bone Texture Alterations in Patients with CKD Using HR-pQCT**

Youngjun Lee *et al.*

*Corresponding author. Dr. Rachel K. Surowiec, Email: [rsurowie@purdue.edu](mailto:rsurowie@purdue.edu)

This PDF File includes:

Details in High-resolution peripheral quantitative computed tomography (HR-pQCT)

Figures. S1 to S6

Tables. S1 to S5

Supplemental References

**1.1. High-resolution peripheral quantitative computed tomography (HR-pQCT)**

HR-pQCT (XtremeCT II, Scanco Medical, Bruttisellen, Switzerland) was acquired on the participants’ non-dominant arm and leg, as previously described (1). Bone length was measured in triplicate using a segmometer (Realmet Flexible Segmometer, NutriActiva, Minneapolis, MN) as described elsewhere (2). Participants were scanned supine on a movable treatment plinth and the limb of interest immobilized using manufacturer-provided carbon fiber casts. The scanner was operated at 68 kVp and 1.47 mA, capturing 168 slices (equivalent to 10.2 mm of bone length) with a voxel size of 60.7 μm. Scanner stability was verified throughout the duration of data collection by scanning phantoms with inserts of known density and volume, per manufacturer instructions. Scan stacks were positioned at 4% and 30% of the bone length proximal to the radius reference line and at 7.3% and 30% of the bone length proximal to the tibia reference line. Scans were reconstructed using manufacturer-supplied algorithms. Once the cortical bone was extracted, the following outcomes were recorded at the diaphyseal and distal site: volumetric BMD (Ct.vBMD, mgHA/cm^3^), area (Ct.Ar, mm^2^), thickness (Ct.Th, mm), and porosity (Ct.Po, %). µFE analysis (Scanco Medical FE software version 1.13) was used to estimate stiffness (kN/mm) and failure load (N) using a pixel-wise assigned modulus of 10 GPa and a Poisson’s ratio of 0.3 and evaluated under axial compression as previously described (3).

**1.1.1 Participant characteristics**

Participant height (measured to the nearest 0.1 cm) and weight (measured to the nearest 0.1 kg) were obtained without shoes, using a calibrated stadiometer (Seca 264; Seca GmbH & Co., Hamburg, Germany) and scale (MS140–300; Brecknell, Fairmont, MN), respectively. Whole-body, total hip, and femoral neck aBMD was assessed using either a Norland Elite (Norland at Swissray, Fort Atkinson, WI) or Hologic Horizon A (Hologic Inc., Bedford, MA, USA) scanner. The manufacturers’ recommended imaging protocols were utilized. Outcomes obtained using the Norland Elite scanner were converted to Hologic Horizon equivalent values using regression formulae derived by scanning 30 individuals on each scanner.

**
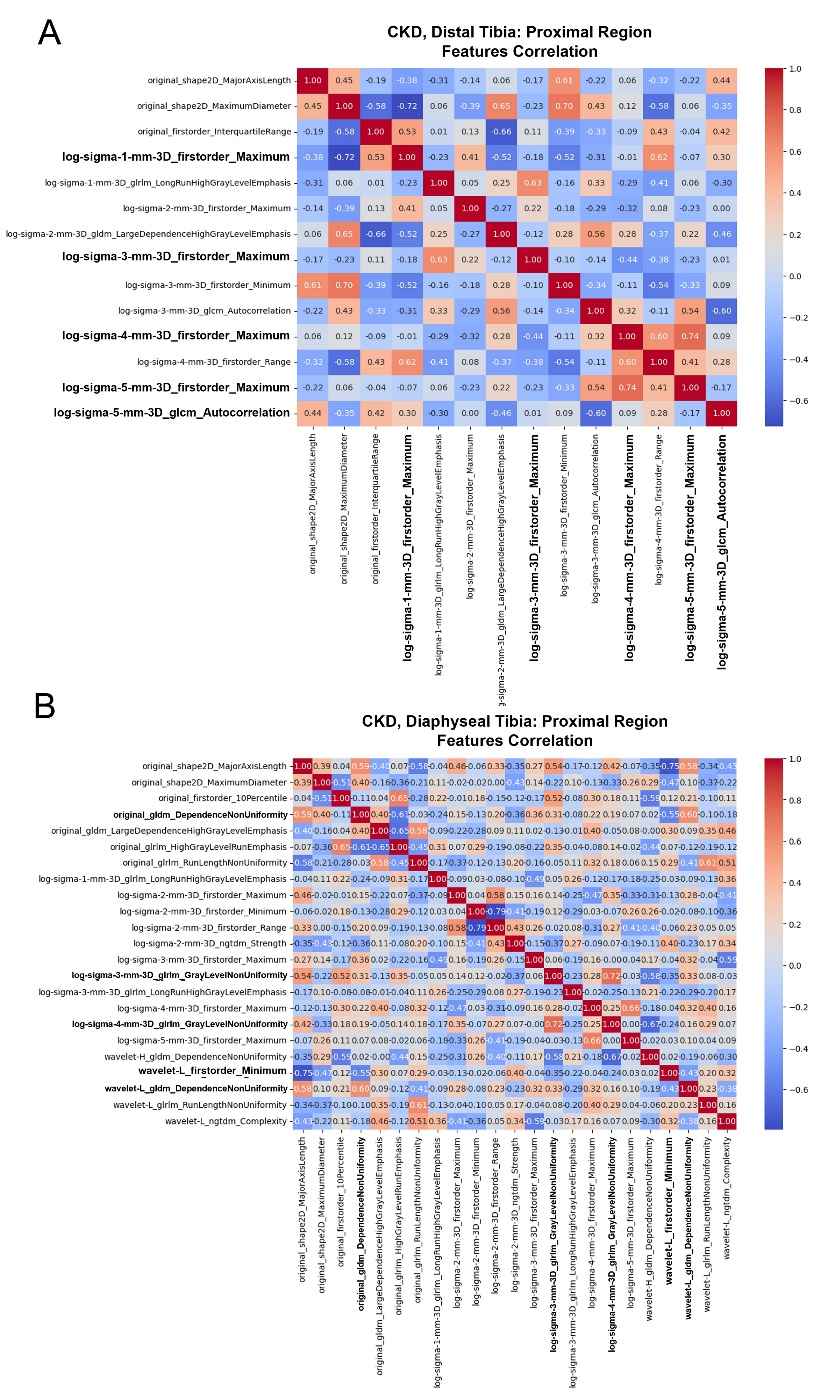
**

**Figure S1**. Comparative correlation analysis of bone texture features in CKD patients: A heatmap visualization of the proximal subregion of the distal and diaphyseal tibia bone sites. The image presents two correlation matrices to present the strength and direction of correlations between different features, showing a visual comparison of how these features correlate differently in the two sites. Bolded are the top 1-5 ranked radiomic features. Proximal region - A: Distal tibia site in CKD, B: Diaphyseal tibia site in CKD.


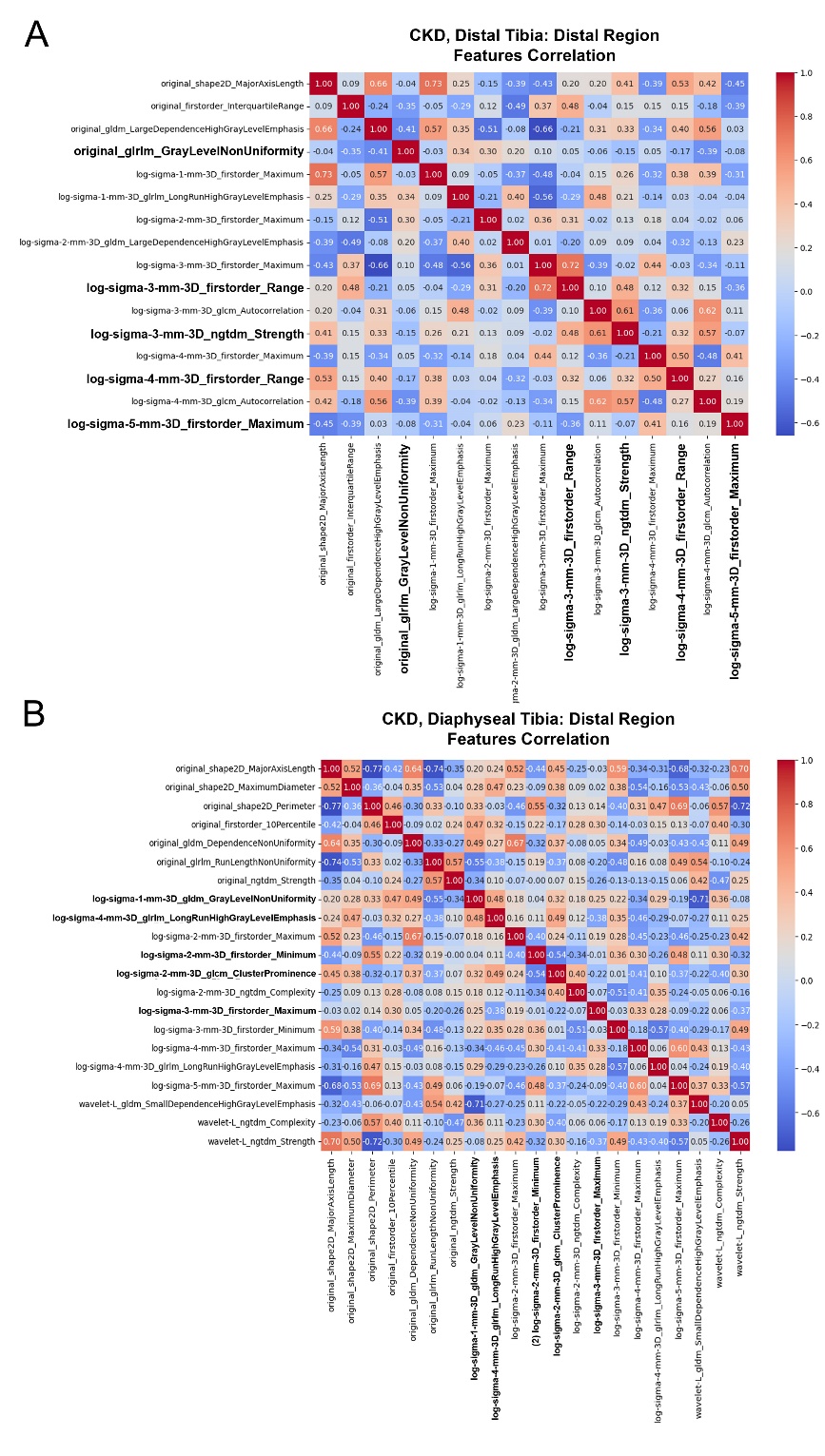


**Figure S2**. Comparative correlation analysis of bone texture features in CKD patients: A heatmap visualization of the distal subregion of the distal and diaphyseal tibia bone sites. The image presents two correlation matrices to present the strength and direction of correlations between different features, showing a visual comparison of how these features correlate differently in the two sites. Bolded are the top 1-5 ranked radiomic features. Distal region - A: Distal tibia site in CKD, B: Diaphyseal tibia site in CKD.

**
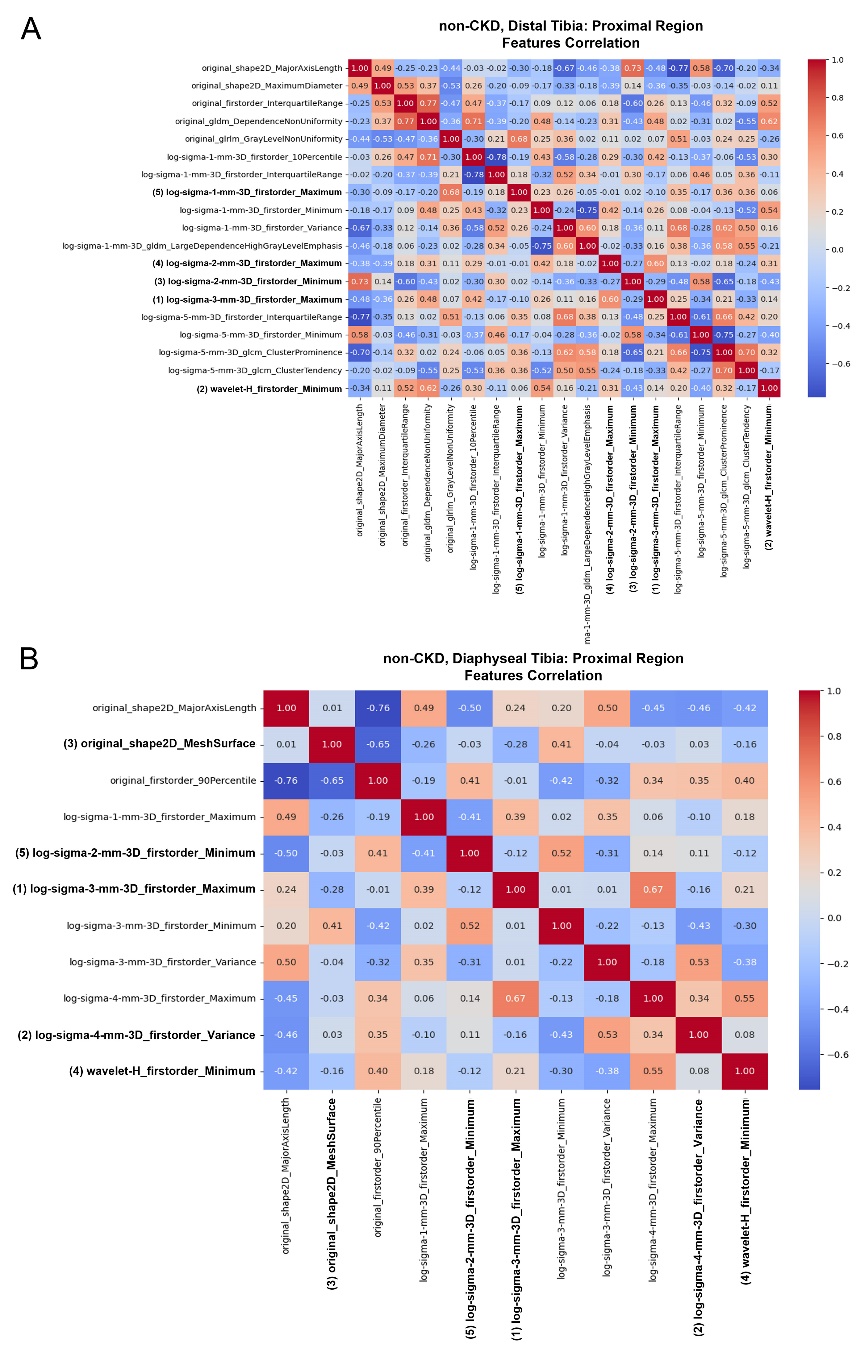
**

**Figure S3**. Comparative correlation analysis of bone texture features in non-CKD volunteers: A heatmap visualization of the proximal subregion of the distal and diaphyseal tibia bone sites. The image presents two correlation matrices to present the strength and direction of correlations between different features, showing a visual comparison of how these features correlate differently in the two sites. Bolded are the top 1-5 ranked radiomic features. Proximal region - A: Distal tibia site in non-CKD, B: Diaphyseal tibia site in non-CKD.


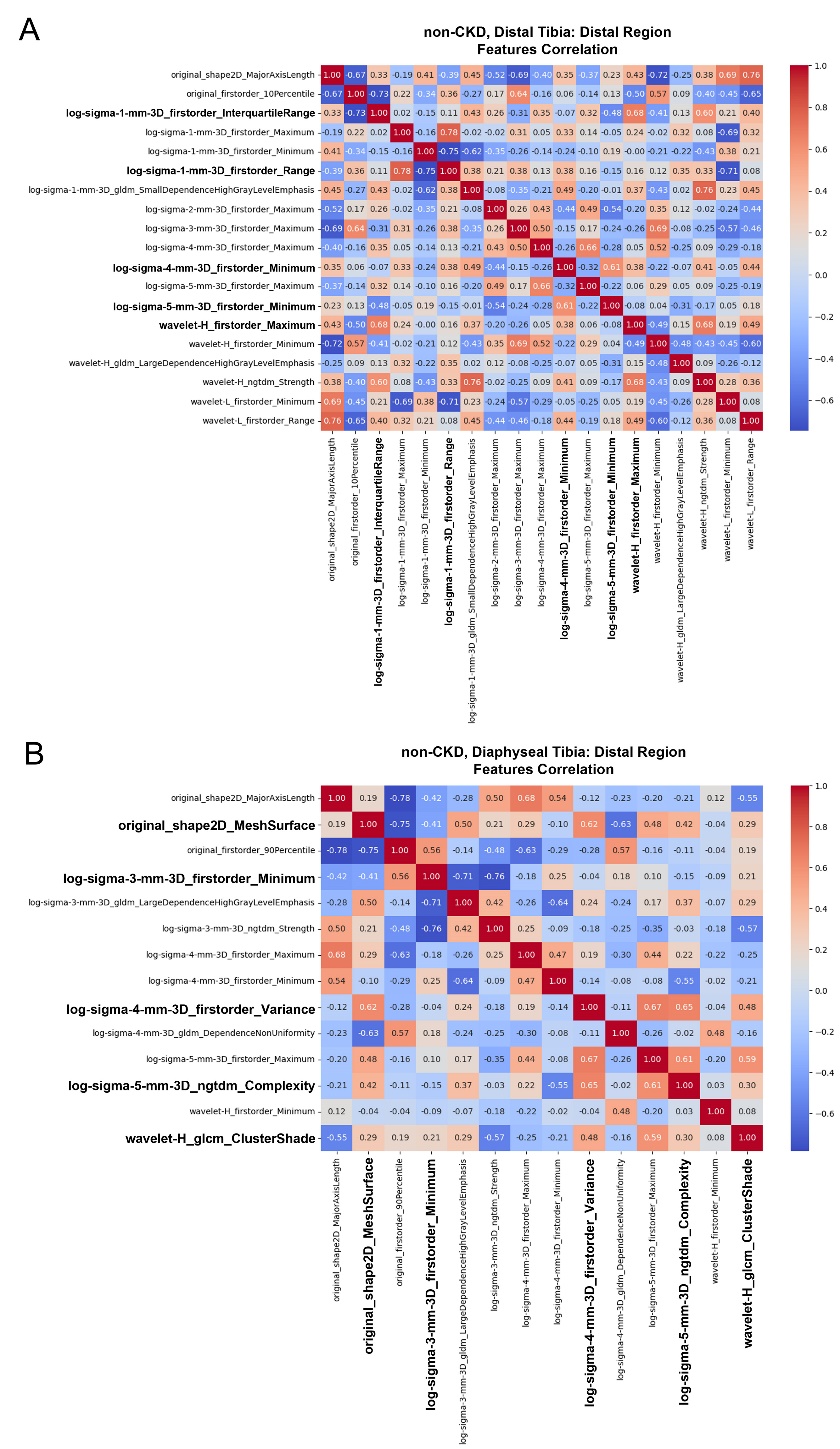


**Figure S4**. Comparative correlation analysis of bone texture features in non-CKD volunteers: A heatmap visualization of the distal subregion of the distal and diaphyseal tibia bone sites. The image presents two correlation matrices to present the strength and direction of correlations between different features, showing a visual comparison of how these features correlate differently in the two sites. Bolded are the top 1-5 ranked radiomic features. Distal region - A: Distal tibia site in non-CKD, B: Diaphyseal tibia site in non-CKD.

**
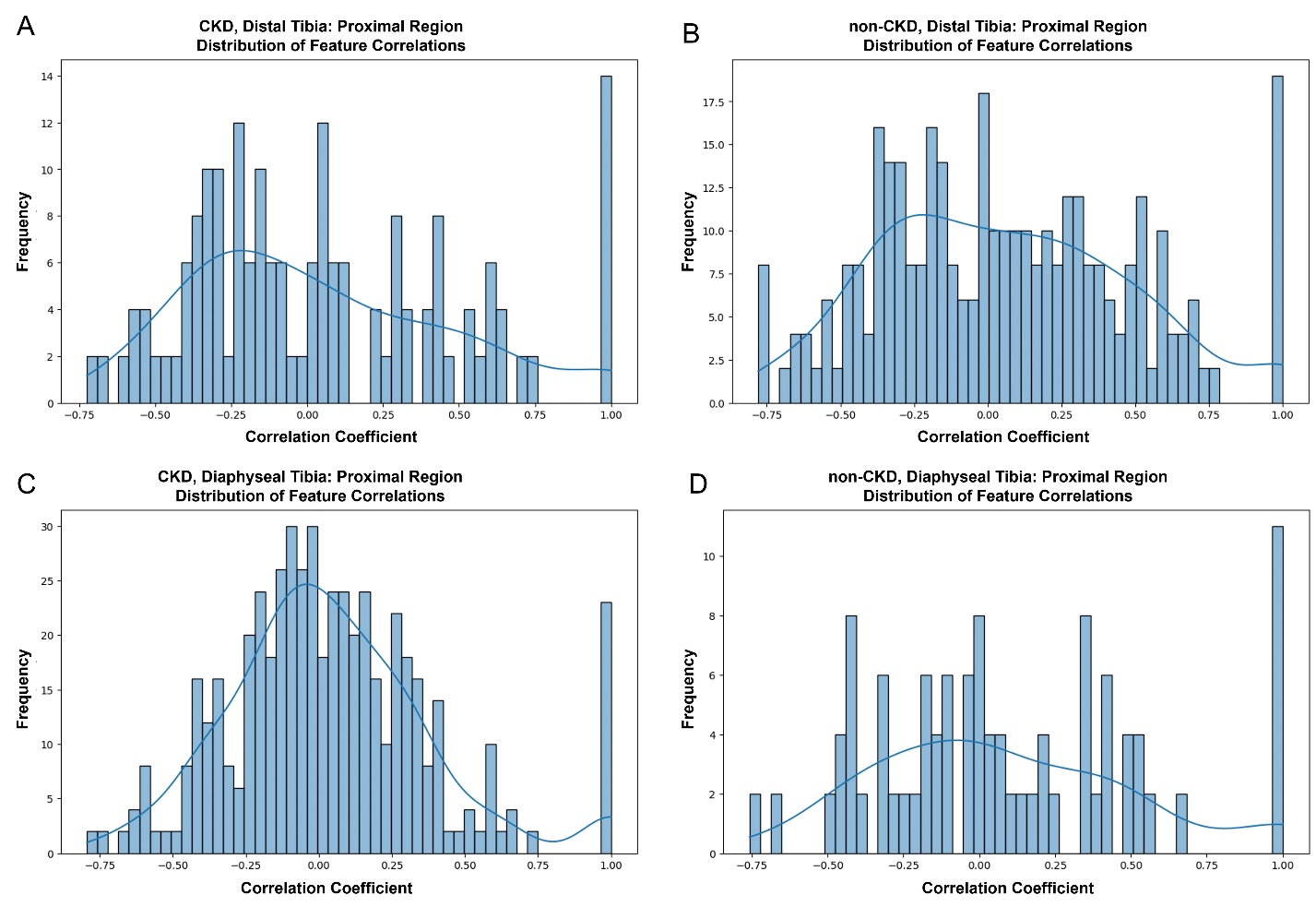
**

**Figure S5**. Distribution of feature correlations in non-CKD and CKD patients: Proximal subregion of distal tibia bone. Both histograms show a distribution of correlation coefficient centered around zero. There is a noticeable spike at a correlation coefficient of 1.0 in both graphs, which represents each feature’s perfect correlation with itself.


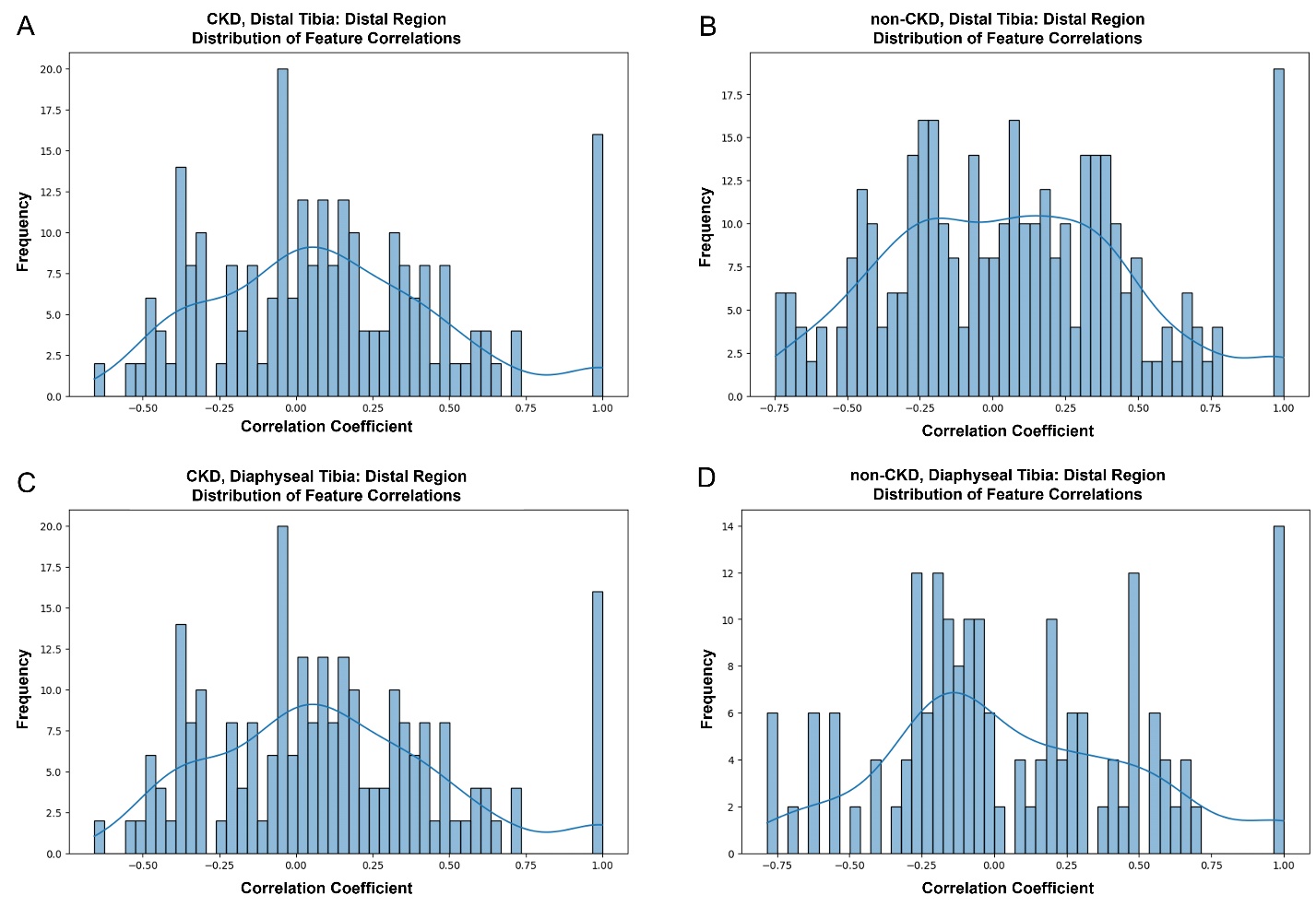


**Figure S6**. Distribution of feature correlations in non-CKD and CKD patients: Distal subregion of distal tibia bone. Both histograms show a distribution of correlation coefficient centered around zero. There is a noticeable spike at a correlation coefficient of 1.0 in all graphs, which represents each feature’s perfect correlation with itself.

**Table S1.** Serum PTH and Vitamin D Levels.

| **Variable** | **CKD (Stage 5D)** |
| --- | --- |
| PTH (pg/mL) ± SEM | 467.2 ± 98.9 |
| VitD (ng/mL) ± SEM | 28.0 ± 2.9 |
| Circulating PTH and 25‑hydroxyvitamin D [25(OH)D] levels were obtained via retrospective chart review and are reported as mean ± SEM; assays were not uniformly timed with HR‑pQCT imaging. | |

**Table S2.** Sex-stratified cortical parameters (cortical area, cortical vBMD, and cortical porosity) at the distal and proximal tibia in non-CKD and CKD groups.

| **Group** | | **Metric** | **Mean ± standard deviation** | |  |
| --- | --- | --- | --- | --- | --- |
|  |  |  | **Female** | **Male** | **P-value** |
| Non-CKD | Distal | Ct.Ar (mm²) | 124.04 ± 21.88 | 159.11 ± 24.18 | 0.002 |
|  |  | Ct.vBMD (mgHA/cm³) | 913.67 ± 67.60 | 868.85 ± 70.35 | 0.083 |
|  |  | Ct.Po (%) | 0.03 ± 0.01 | 0.03 ± 0.02 | 0.227 |
|  | Proximal | Ct.Ar (mm²) | 252.80 ± 31.40 | 331.89 ± 37.92 | 0.001 |
|  |  | Ct.vBMD (mgHA/cm³) | 1037.94 ± 27.51 | 1019.76 ± 17.08 | 0.152 |
|  |  | Ct.Po (%) | 0.01 ± 0.00 | 0.01 ± 0.01 | 0.216 |
| CKD | Distal | Ct.Ar (mm²) | 144.02 ± 33.78 | 116.82 ± 15.66 | 0.016 |
|  |  | Ct.vBMD (mgHA/cm³) | 844.24 ± 92.01 | 886.23 ± 95.54 | 0.250 |
|  |  | Ct.Po (%) | 0.03 ± 0.02 | 0.03 ± 0.02 | 0.213 |
|  | Proximal | Ct.Ar (mm²) | 308.32 ± 58.54 | 246.33 ± 39.43 | 0.005 |
|  |  | Ct.vBMD (mgHA/cm³) | 1004.34 ± 49.93 | 1022.79 ± 38.48 | 0.312 |
|  |  | Ct.Po (%) | 0.01 ± 0.01 | 0.01 ± 0.00 | 0.334 |

**Table S3.** Effect of variance and inter-correlation thresholds on retained feature counts in representative proximal and distal tibial regions.

| **Region** | **Variance** | | | | | **Correlation** | | | | |
| --- | --- | --- | --- | --- | --- | --- | --- | --- | --- | --- |
|  | **<0.1** | **<0.2** | **<0.3** | **<0.4** | **<0.5** | **>0.9** | **>0.8** | **>0.7** | **>0.6** | **>0.5** |
| Distal Tibia Prox. (Non-CKD) | 480 | 354 | 58 | 22 | 9 | 45 | 19 | 6 | 2 | 0 |
| Distal Tibia Prox. (CKD) | 490 | 375 | 61 | 25 | 11 | 52 | 14 | 5 | 2 | 0 |
| Diaphyseal Tibia Prox. (Non-CKD) | 460 | 314 | 49 | 18 | 7 | 40 | 11 | 4 | 1 | 1 |
| Diaphyseal Tibia Prox. (CKD) | 495 | 373 | 66 | 28 | 12 | 60 | 23 | 7 | 2 | 0 |
| Distal Tibia Dist. (Non-CKD) | 478 | 380 | 55 | 20 | 8 | 44 | 19 | 4 | 2 | 0 |
| Distal Tibia Dist. (CKD) | 488 | 373 | 60 | 24 | 10 | 50 | 16 | 8 | 3 | 0 |
| Diaphyseal Tibia Dist. (Non-CKD) | 459 | 313 | 52 | 19 | 7 | 41 | 14 | 5 | 2 | 0 |
| Diaphyseal Tibia Dist. (CKD) | 494 | 374 | 65 | 27 | 11 | 59 | 21 | 6 | 2 | 0 |

Note: Values represent the number of radiomic features retained after sequential filtering by variance thresholds (<0.1–0.5) and inter-correlation thresholds (>0.9–0.5). Results are shown for representative proximal regions of the distal and diaphyseal tibia in CKD and non-CKD groups. Variance thresholds above 0.3 or correlation thresholds stricter than 0.8 markedly reduced the retained feature set, often to fewer than 10–20 features, supporting the use of variance <0.2 and correlation >0.8 as optimized thresholds for stability and performance.

**Table S4.** Correlation of top radiomic features with finite element outcomes of the non-CKD group in the proximal/distal subregion of the distal/diaphyseal tibia site.

| **Bone** | **Location** | **Feature** | **Finite Element (**$\mu$**FE)** | **Correlation**  **(95% CI)** | **P-value** |
| --- | --- | --- | --- | --- | --- |
| Distal | Distal | Strength (NGTDM) | Ct.Stiff.dist (kN/mm) | -0.128 [-0.438, 0.209] | 0.444 |
|  |  |  | Ct.Fload.dist (kN) | 0.110 [-0.227, 0.423] | 0.511 |
|  |  | Minimum  (First order) | Ct.Stiff.dist (kN/mm) | 0.040 [-0.292, 0.364] | 0.810 |
|  |  |  | Ct.Fload.dist (kN) | -0.030, [-0.355, 0.302] | 0.859 |
|  | Proximal | Strength  (NGTDM) | Ct.Stiff.prox (kN/mm) | 0.031, [-0.301, 0.356] | 0.855 |
|  |  |  | Ct.Fload.prox (kN) | -0.048 [-0.371, 0.285] | 0.775 |
|  |  | Minimum  (First order) | Ct.Stiff.prox (kN/mm) | -0.116 [-0.428, 0.221] | 0.488 |
|  |  |  | Ct.Fload.prox (kN) | 0.107 [-0.230, 0.420] | 0.524 |
| Diaphyseal | Distal | Strength (NGTDM) | Ct.Stiff.dist (kN/mm) | -0.996 [-0.255, 0.433] | 0.569 |
|  |  |  | Ct.Fload.dist (kN) | -0.110 [-0.441, 0.246] | 0.534 |
|  |  | Minimum  (First order) | Ct.Stiff.dist (kN/mm) | -0.197 [-0.509, 0.162] | 0.265 |
|  |  |  | Ct.Fload.dist (kN) | 0.196 [-0.162, 0.509] | 0.267 |
|  | Proximal | Strength  (NGTDM) | Ct.Stiff.prox (kN/mm) | -0.297 [-0.584, 0.056] | 0.088 |
|  |  |  | Ct.Fload.prox (kN) | 0.275 [-0.079, 0.568] | 0.115 |
|  |  | Minimum  (First order) | Ct.Stiff.prox (kN/mm) | 0.072 [-0.282, 0.409] | 0.685 |
|  |  |  | Ct.Fload.prox (kN) | -0.075 [-0.411, 0.280] | 0.675 |

Note: $P$ < 0.05 means statistical significance. µFE outcomes of stiffness and failure load considered cortical bone compartments. Ct.Stiff.dist = distal total stiffness; Ct.Fload.dist = distal total failure load; Ct.Stiff.prox (kN) = proximal total stiffness; Ct.Fload.prox (kN) = proximal total failure load.

**Table S5.** Correlation of top radiomic features with finite element outcomes of the CKD group in the proximal/distal subregion of the distal/diaphyseal tibia site.

| **Bone** | **Location** | **Feature** | **Finite Element (**$\mu$**FE)** | **Correlation**  **(95% CI)** | **P-value** |
| --- | --- | --- | --- | --- | --- |
| Distal | Distal | Strength (NGTDM) | Ct.Stiff.dist (kN/mm) | -0.142 [-0.471, 0.221] | 0.429 |
|  |  |  | Ct.Fload.dist (kN) | 0.123 [-0.240, 0.456] | 0.496 |
|  |  | Minimum  (First order) | Ct.Stiff.dist (kN/mm) | 0.174 [-0.190, 0.496] | 0.333 |
|  |  |  | Ct.Fload.dist (kN) | -0.153 [-0.480, 0.211] | 0.396 |
|  | Proximal | Strength  (NGTDM) | Ct.Stiff.prox (kN/mm) | -0.081 [-0.422, 0.279] | 0.653 |
|  |  |  | Ct.Fload.prox (kN) | 0.084 [-0.277, 0.424] | 0.644 |
|  |  | Minimum  (First order) | Ct.Stiff.prox (kN/mm) | 0.132 [-0.231, 0.463] | 0.464 |
|  |  |  | Ct.Fload.prox (kN) | -0.132 [-0.463, 0.232] | 0.465 |
| Diaphyseal | Distal | Strength (NGTDM) | Ct.Stiff.dist (kN/mm) | 0.059 [-0.339, 0.439] | 0.772 |
|  |  |  | Ct.Fload.dist (kN) | -0.036 [-0.420, 0.359] | 0.858 |
|  |  | Minimum  (First order) | Ct.Stiff.dist (kN/mm) | -0.168 [-0.524, 0.237] | 0.401 |
|  |  |  | Ct.Fload.dist (kN) | 0.159 [-0.246, 0.517] | 0.427 |
|  | Proximal | Strength  (NGTDM) | Ct.Stiff.prox (kN/mm) | 0.092 [-0.310, 0.465] | 0.650 |
|  |  |  | Ct.Fload.prox (kN) | -0.084 [-0.459, 0.316] | 0.676 |
|  |  | Minimum  (First order) | Ct.Stiff.prox (kN/mm) | 0.004 [-0.386, 0.394] | 0.983 |
|  |  |  | Ct.Fload.prox (kN) | -0.005 [-0.394, 0.386] | 0.981 |

Note: $P$ < 0.05 means statistical significance. µFE outcomes of stiffness and failure load considered cortical bone compartments. Ct.Stiff.dist = distal total stiffness; Ct.Fload.dist = distal total failure load; Ct.Stiff.prox (kN) = proximal total stiffness; Ct.Fload.prox (kN) = proximal total failure load.

**Supplemental References**

1. Warden SJ, Liu Z, Fuchs RK, van Rietbergen B, Moe SM. Reference data and calculators for second-generation HR-pQCT measures of the radius and tibia at anatomically standardized regions in White adults. Osteoporosis International. 2022:1-16.

2. Bonaretti S, Majumdar S, Lang TF, Khosla S, Burghardt AJ. The comparability of HR-pQCT bone measurements is improved by scanning anatomically standardized regions. Osteoporosis International. 2017;28:2115-28.

3. Arias-Moreno AJ, Hosseini HS, Bevers M, Ito K, Zysset P, van Rietbergen B. Validation of distal radius failure load predictions by homogenized-and micro-finite element analyses based on second-generation high-resolution peripheral quantitative CT images. Osteoporosis international. 2019;30:1433-43.
